# Function and solution structure of the Arabidopsis thaliana RALF8 peptide

**DOI:** 10.1002/pro.3628

**Published:** 2019-05-13

**Authors:** Ronnie O. Frederick, Miyoshi Haruta, Marco Tonelli, Woonghee Lee, Gabriel Cornilescu, Claudia C. Cornilescu, Michael R. Sussman, John L. Markley

**Affiliations:** ^1^ National Magnetic Resonance Facility at Madison University of Wisconsin‐Madison Madison Wisconsin 53706; ^2^ Biotechnology Center University of Wisconsin‐Madison Madison Wisconsin 53706; ^3^ Department of Biochemistry University of Wisconsin‐Madison Madison Wisconsin 53706

**Keywords:** rapid alkanization factor (RALF), peptide structure, NMR solution structure, peptide production, stable isotope labeling, disulfide pairing, root growth assay, cytoplasmic calcium activation assay, peptide dynamics

## Abstract

We report the recombinant preparation from Escherichia coli cells of samples of two closely related, small, secreted cysteine‐rich plant peptides: rapid alkalinization factor 1 (RALF1) and rapid alkalinization factor 8 (RALF8). Purified samples of the native sequence of RALF8 exhibited well‐resolved nuclear magnetic resonance (NMR) spectra and also biological activity through interaction with a plant receptor kinase, cytoplasmic calcium mobilization, and *in vivo* root growth suppression. By contrast, RALF1 could only be isolated from inclusion bodies as a construct containing an N‐terminal His‐tag; its poorly resolved NMR spectrum was indicative of aggregation. We prepared samples of the RALF8 peptide labeled with ^15^N and ^13^C for NMR analysis and obtained near complete ^1^H, ^13^C, and ^15^N NMR assignments; determined the disulfide pairing of its four cysteine residues; and examined its solution structure. RALF8 is mostly disordered except for the two loops spanned by each of its two disulfide bridges.

## Abstract 2

PDB Code(s): 6NU4;

## Introduction

The small (~5 kDa), secreted, basic peptides RALF1 and RALF8 are members of the rapid alkalinization factor (RALF) family of plant peptides that affect plant growth, pathogen response, and pollen tube generation.^1^, ^2^, ^3^, ^4^, ^5^ The importance of the family of RALF peptides in plants is supported by the presence of a large number of genomic DNA sequences encoding RALF and RALF‐like peptides. A recent study of computationally identified RALF‐like sequences describes 795 RALF coding sequences in 51 different plant species.^6^ There are around 36 known members of this family of peptides in *Arabidopsis*, but little information is available on their three‐dimensional (3D) structure despite their important roles in cell signaling and numerous critical plant development processes.^7^ Genome‐wide gene expression profiling reveals that each RALF member is characterized by tissue‐ and development‐specific expression patterns. For example, RALF1 is most highly expressed in root and acts as a negative regulator of root growth.^8^ On the other hand, RALF4 is preferentially expressed in pollen and regulates pollen tube growth.^2^, ^9^ RALF peptides are shown to act as growth regulatory factors that suppress growth by receptor‐mediated rapid events involving protein phosphorylation, cytoplasmic calcium elevation, and extracellular pH alkalinization.^1^ A family of CrRLKs (*Catharanthus roseus* receptor‐like kinases) consisting of 17 members in *Arabidopsis* is known to comprise receptors for RALF peptides,^2^, ^5^, ^10^ consistent with a model that CrRLKs are the receptors for RALFs. *Arabidopsis* knockout mutants of CrRLKs, including FERONIA receptor (FER), lack cellular responses to RALF‐induced rapid cytoplasmic calcium elevation. Thus, the measurement of cytoplasmic calcium changes has been used as an assay for examining cellular sensitivity to this peptide ligand.

The sequence identity among the RALF peptides varies significantly, but they contain four highly conserved cysteine residues that have been shown to be required for biological activity.^11^ RALF1, which is the predominant RALF isoform present in *Arabidopsis thaliana* seedlings, suppresses root elongation via FER‐mediated protein phosphorylation signaling, and this interaction *in vivo* most likely involves other interacting proteins or chaperones.^8^, ^10^, ^12^, ^13^, ^14^, ^15^ A recent *in vitro* crosslink study indicated that the C‐terminal region of RALF1 can be colocalized with the amino terminal region of FER, within the ~11 Å chemical crosslinking distance.^16^ RALF8's involvement in root growth was reported in a study examining the transcriptomic responses of plants that were exposed to simultaneous water deficit and nematode stresses. A subsequent phenotyping assay with RALF8‐overexpressing plants further demonstrated its role in root growth.^17^

**Figure 1:**
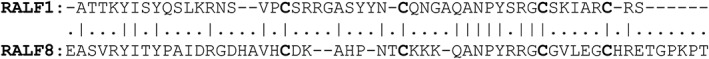
Aligned amino acid sequences of the RALF1 and RALF8 peptides. The four conserved cysteines are highlighted in bold (Smith–Watermann algorithm). The two peptides share about 34% sequence identity.

In preliminary tests of *Escherichia coli* expression of several RALF peptides of known biological function, we were successful in producing two peptides, namely RALF1 and RALF8. Figure 1 shows a comparison of the aligned amino acid sequences of the secreted RALF1 and RALF8 peptides. The RALF peptide family contains distinctive sequence features including the presence of four conserved cysteine residues, an abundance of basic amino acids, and a conserved YISY motif in the amino terminal region of the peptide.^18^, ^19^ RALF1 (pI = 10.06) contains no acidic residues, no histidine residues, and one proline, whereas RALF8 (pI = 9.08) contains three acidic residues, four histidine residues, and four proline residues; this suggests that the two RALF peptides likely have distinctive side chain structures when exposed to different pH environments. We successfully produced biologically active recombinant RALF8 peptides from *E. coli* strains, and then used an *in vivo* assay based on inhibition of plant root growth to demonstrate that the peptide regulates root growth through a FER signaling pathway.^20^ By contrast, RALF1 samples produced from *E. coli* under a number of expression, isolation, and purification conditions (including those successful with RALF8) exhibited aggregation, suggesting a non‐native state and yielded poor quality nuclear magnetic resonance (NMR) spectra. RALF8 yielded well‐resolved NMR spectra, and we were able to determine its structure in solution. Our results show that RALF8 is largely disordered with the exception of two ordered loops, each spanned by a disulfide bridge.

## Results and Discussion

**Figure 2:**
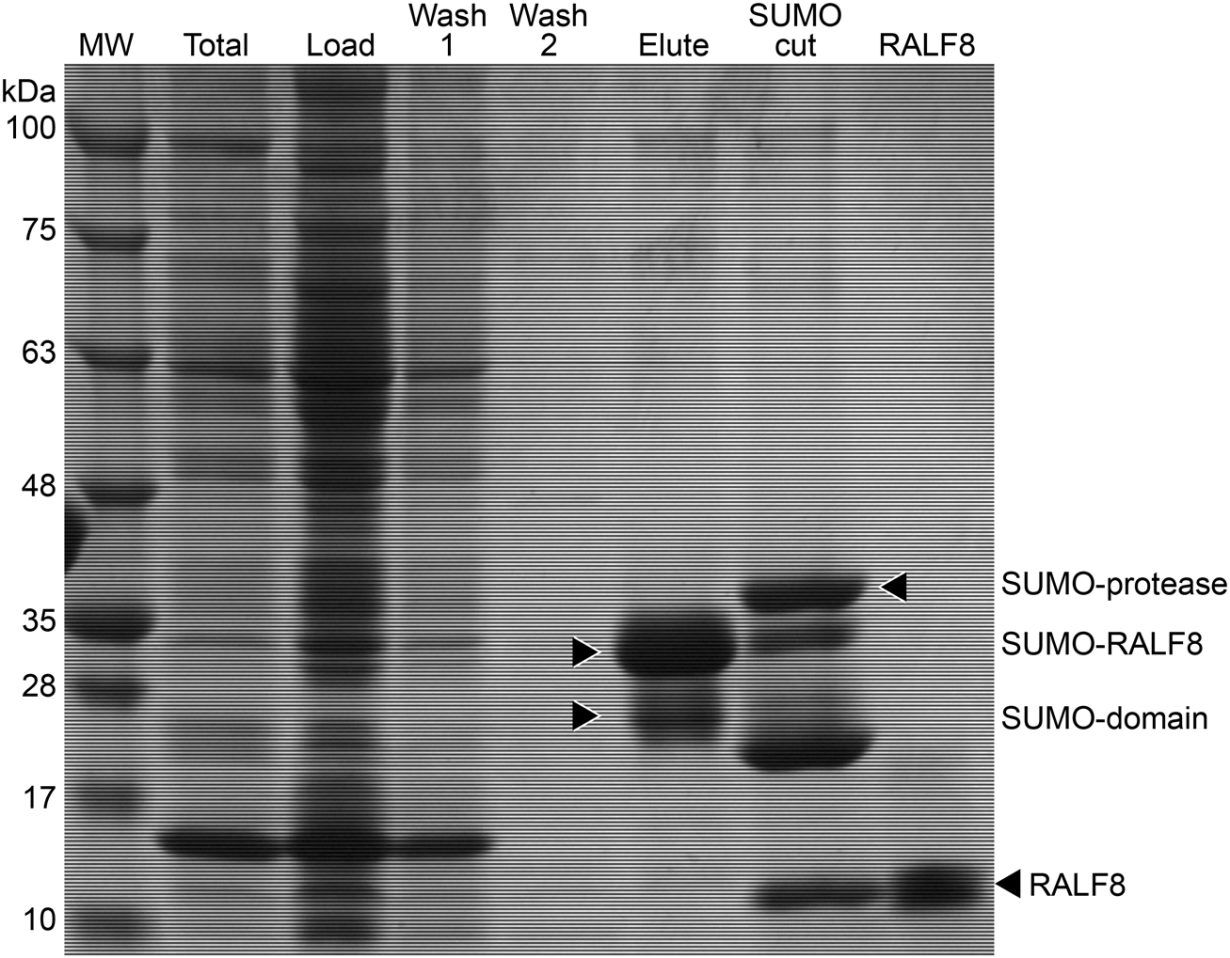
Sodium dodecly sulfate ‐ polyacrylamide gel electrophoresis (SDS‐PAGE) analysis of steps in the production and purification of RALF8. “TOTAL” is from cell lysate. “LOAD” represents the material loaded onto the IMAC column. “WASH 1” and “WASH 2” are from successive column washes. “ELUTE” is from the eluate. “SUMO CUT” is the sample following the addition of SUMO protease. “RALF8” is from the purified protein. The final yield of purified RALF8 was usually 3.5 mg/L minimal medium.

**Figure 3:**
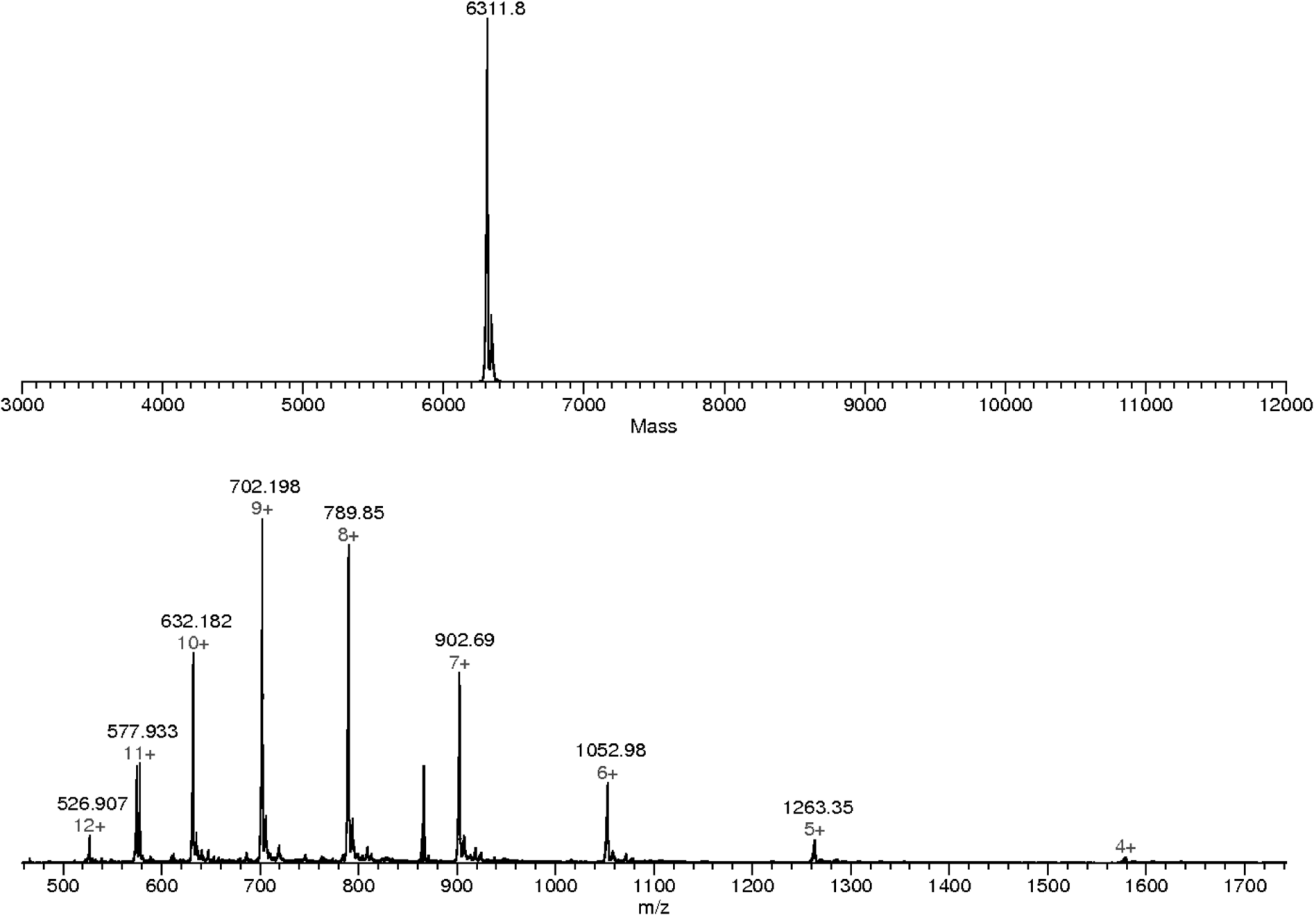
Characterization of ^15^N‐labeled RALF8 by ESI‐TOF mass spectrometry. (Top panel) Reconstructed mass of ^15^N‐labeled RALF8. (Bottom panel) Peptide ions showing multiple charged species ranging from *m*/*z* 1263.35 (5+) to 526.907 (12+).

The genes coding for RALF1 and RALF8 was cloned from *A. thaliana*, and the peptides corresponding to excreted peptides were produced from *E. coli* cells. Of the many approaches tried with RALF1, the construct with the highest yield used the Flexi vector pVP67K with an N‐terminal His‐tag.^21^ However, most of the RALF1 peptides produced was in insoluble inclusion bodies and resisted refolding. By contrast, we were able to prepare the native sequence of RALF8 as a soluble active peptide using the pE‐SUMO (small ubiquitin modifier) (Kan) vector.^22^ Figure 2 shows sodium dodecly sulfate ‐ polyacrylamide gel electrophoresis (SDS‐PAGE) analyses at various steps in the isolation and purification of RALF8. The mass spectrum of the ^15^N‐labeled RALF8 peptide (Fig. 3) shows that the level of isotopic enrichment was about 93%. These results also indicate that the peptide is the full length expected.

**Figure 4:**
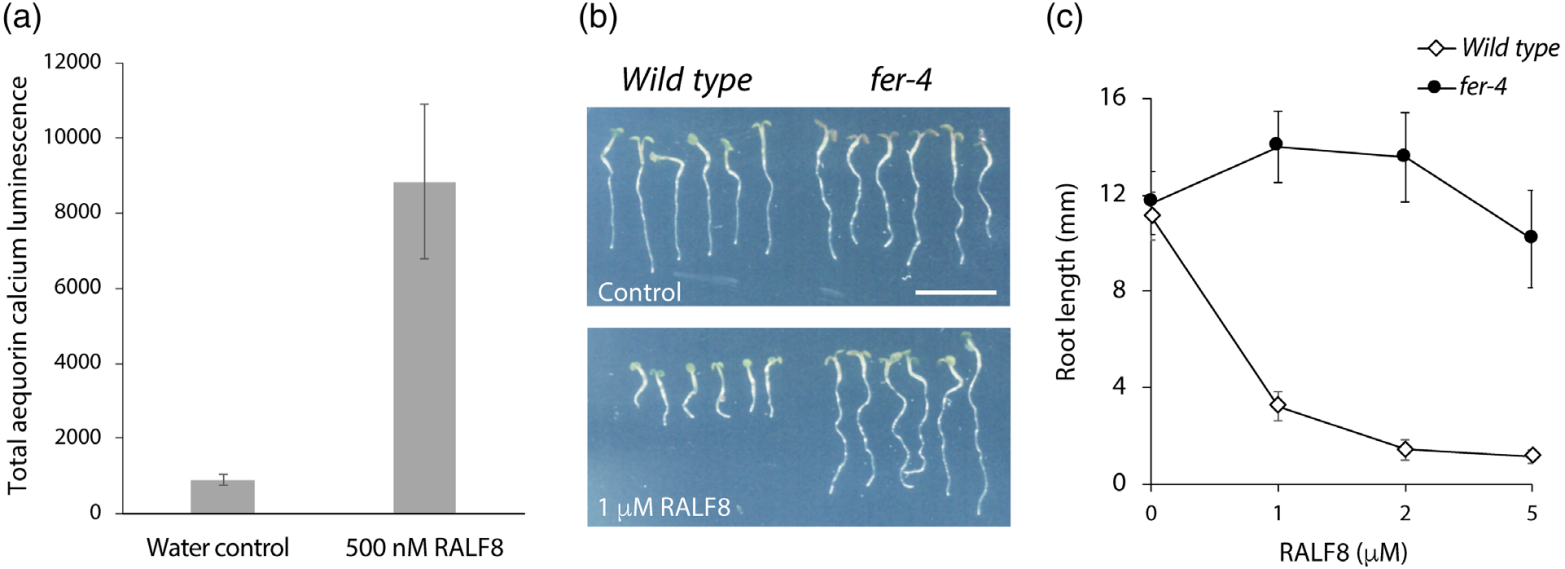
Biological assays of the RALF8 peptide. (a) Cytoplasmic calcium mobilization activity of the RALF8 peptide. (b) Image of Arabidopsis thaliana seedlings grown in the presence of RALF8. Three‐day‐old germinating seedlings were grown in the absence (top panel) or presence (bottom panel) of 1 μ*M* RALF8 peptide for 2 days. FERONIA receptor kinase mutant (*fer‐4*) showed insensitivity to RALF8‐caused inhibition compared with the wild type. (c) Dose–response curve of RALF8 inhibitory effects on the wild type and *fer‐4* mutant seedlings. Data are shown as the mean ± SD, *n* = 6.

To examine the *in vivo* activity of the isolated RALF8 peptide, we have tested both a rapid response, cytoplasmic calcium elevation and a long‐term effect, root growth suppression of *Arabidopsis* seedlings. RALF8 samples were shown to be active by a cytoplasmic calcium mobilization assay [Fig. 4(a)] and by a root growth inhibition assay [Fig. 4(b,c)]. Because root growth suppression caused by the RALF1 peptide involves the function of a plasma membrane receptor, FERONIA,^10^ we also examined RALF8‐caused root growth suppression with a *feronia* (*fer*) knockout mutant plant. Wild‐type roots showed 75% growth inhibition at 1 μ*M* RALF8 concentration compared with the control condition, whereas *fer* knockout mutant roots were completely insensitive to RALF8‐caused suppression up to 5 μ*M* RALF8, with the highest concentration examined in this study. This observation indicates that FER either directly or indirectly mediates RALF8‐induced signaling downstream events leading to root growth suppression. The involvement of FER in cellular responses to RALF isoforms other than RALF1 was also observed in previous studies of RALF4, RALF19, RALF23, and RALF34.^2^, ^5^, ^23^ Further molecular, genetic, and biochemical experiments examining how different isoforms of RALFs and CrRLKs including FER interact to transmit downstream signaling are currently underway in our laboratories.

**Figure 5:**
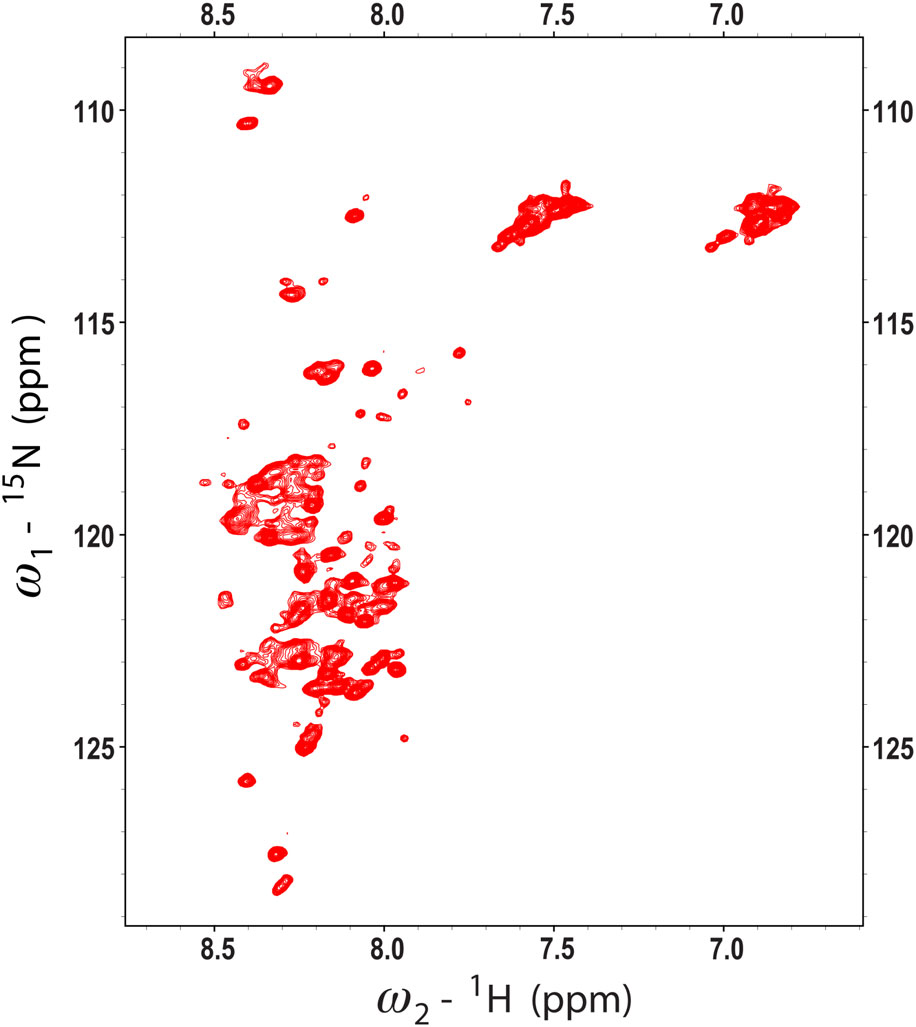
2D ^1^H,^15^N HSQC spectrum of [U‐^15^N]‐RALF1 collected at 600 MHz (^1^H). The lack of dispersion in the ^1^H dimension and broad peaks indicate that the peptide is unstructured and aggregated.

**Figure 6:**
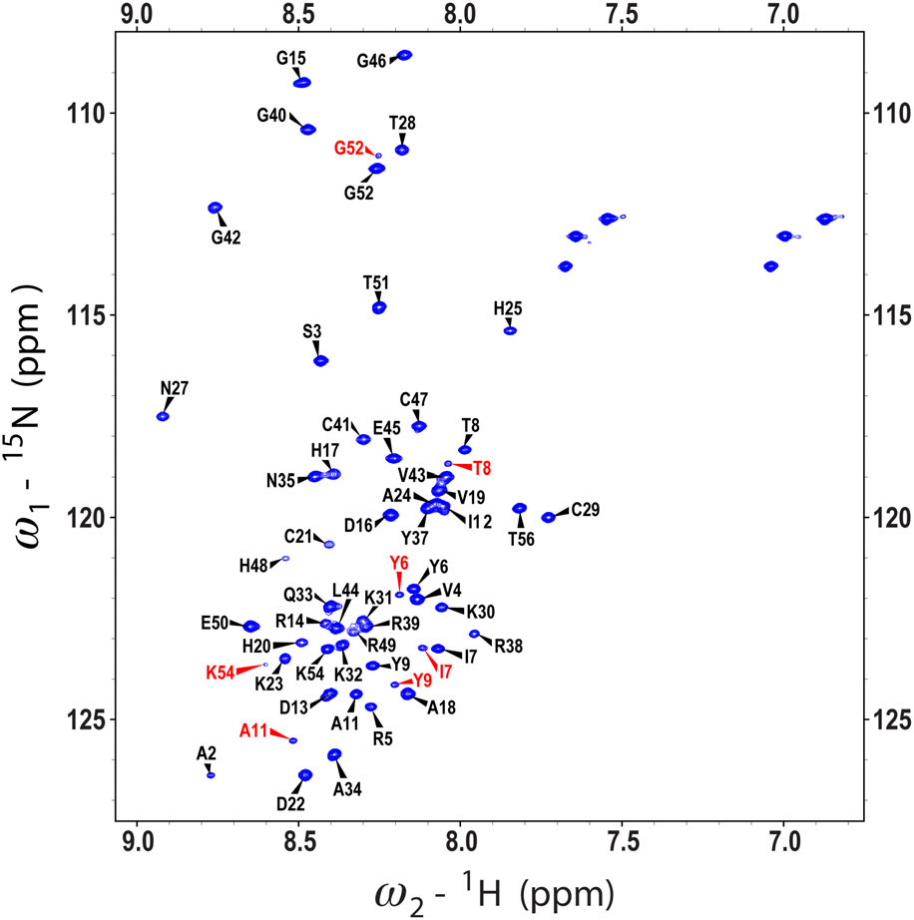
2D ^1^H,^15^N HSQC spectrum of [U‐15N]‐RALF8 collected at 800 MHz (^1^H). Peaks are labeled with the backbone assignments obtained by I‐PINE, followed by manual refinement in NMRFAM‐SPARKY. Doubled backbone peaks (labeled in red) were assigned to several amino acid residues.

The 2D ^1^H,^15^N‐HSQC NMR spectrum of refolded RALF1 at pH 6 (Fig. 5) was poorly dispersed and contained broad peaks. The spectral quality was not improved under different solution conditions, including different salt concentrations and pH values. The poor spectral quality prevented the NMR structural analysis of RALF1, and as a consequence, we turned to RALF8, another variant studied in our laboratory. The peaks in the 2D ^1^H,^15^N‐HSQC spectrum of RALF8 at pH 6 (Fig. 6) were spread across a narrow range of chemical shifts in the ^1^H dimension, suggesting that the protein is highly dynamic in solution. Nevertheless, we achieved near complete chemical shift assignments for RALF8 (all backbones except for those of the first residue (E1), all aliphatic side chains, but no aromatic side chains). A number of residues exhibited evidence of multiple backbone amide peaks, suggesting that at least parts of the protein may populate two distinct conformations. In particular, we were able to identify distinct pairs of backbone amide peaks for residues Y6, I7, T8, Y9, A11, G52, and K54 (labeled in red in Fig. 6). Given the proximity of these residues to two of the proline residues (P10 and P53), this suggested that the peptidyl–prolyl peptide bonds of these two residues may populate both the *trans* and *cis* configurations. Because the peptide is believed to function at a lower pH, we also collected a 2D ^1^H,^15^N‐HSQC NMR spectrum of RALF8 at pH 4.5. Although signals from several residues, in particular histidine residues, exhibited chemical shift differences, the overall pattern was the same. The peptide did not become more structured, and the relative intensities of the major and minor peaks remained the same.

To analyze the question of heterogeneity of peptidyl–prolyl peptide bonds, we examined nuclear Overhauser enhancement (NOE) cross peak data to differentiate signals from *cis* and *trans* configurations and utilized relatively peak intensities from the major and minor signals from individual residues to determine the *trans* and *cis* populations. A strong NOE cross peak was observed between major peaks Y9 ^1^H^α^ and P10 ^1^H^δ^, but not between major peaks Y9 ^1^H^α^ and P10 ^1^H^α^; these findings indicate that the major form has a *trans* Y9–P10 peptide bond. For the corresponding minor form, we found peaks corresponding to ^1^H^N^ of Y6–Y9 and A11, but were unable to locate cross peaks between Y9 ^1^H^α^ and P10 ^1^H^α^ because the latter is close to the water signal and occluded by noise. From the assigned ^1^H^N^ of A11, and with the aid of data from HNCACB, CBCACONH, CCONH, HBHACONH, ^1^H,^13^C‐HSQC, and HCCH‐TOCSY spectra, we identified P10 ^13^C^β^ and ^13^C^γ^ signals from the major and minor forms (Table 1). The Δ(δ^13^C^β^–δ^13^C^γ^) values were consistent with the major form being *trans* and the minor form being *cis*. On the basis of the relative intensities of the major and minor ^1^H^N^ peaks of Y6–Y9 and A11, we calculated Y9–P10 *trans*:*cis* as 74:26.

**Table 1:** ^13^C^β^ and ^13^C^γ^ Chemical Shifts of P10 and P53 in Their Major and Minor Peptide Bond Configurations

Proline residue	Atom(s)	Major form (ppm)	Minor form (ppm)
10	^13^C^β^	32.11	34.08
	^13^C^γ^	27.37	24.79
	Δ(δ^13^C^β^–δ^13^C^γ^)	4.74	9.29
53	^13^C^β^	32.2	34.66
	^13^C^γ^	27.16	24.84
	Δ(δ^13^C^β^–δ^13^C^γ^)	5.04	9.82

For analysis of the G52–P53 peptide bond, we observed an NOE cross peak between major peaks G52 ^1^H^α^ and P53 ^1^H^δ^, but not between major peaks G52 ^1^H^α^ and P53 ^1^H^α^; these findings indicate that the major form has a *trans* G52–P53 peptide bond. From the assigned ^1^H^N^ of K54, and with the aid of data from HNCACB, CBCACONH, CCONH, HBHACONH, ^1^H,^13^C‐HSQC, HCCH‐TOCSY, and NOESY spectra, we identified P53 ^13^C^β^ and ^13^C^γ^ signals from the major and minor forms (Table 1). The Δ(δ^13^C^β^–δ^13^C^γ^) values were consistent with the major form being *trans* and the minor form being *cis*. On the basis of the relative intensities of the major and minor ^1^H^N^ peaks of G52 and K54, we calculated G52–P53 *trans*:*cis* as 85:15.

For analysis of the K54–P56 peptide bond, we observed an NOE cross peak between major peaks K54 ^1^H^α^ and P56 ^1^H^δ^, but not between major peaks K54 ^1^H^α^ and P56 ^1^H^α^; these findings indicate that the major form has a *trans* K54–P56 peptide bond. Because no minor peaks corresponding to P55 or T56 were observed, we conclude that the K54–P56 peptide bond is largely *trans*.

The ^13^C^β^ chemical shifts of the four cysteine residues (C21, C29, C41, and C47) ranged between 40.384 and 43.219 ppm, suggesting that all four are involved in S–S bonds.^24^ To establish the disulfide bond configuration, we looked for ^1^H^β^–^1^H^β^ NOE contacts between disulfide‐bonded cysteine residues in the spectra recorded using a modified ^1^H,^13^C‐HSQC‐NOESY‐^1^H,^13^C‐HSQC pulse program. This NOESY experiment was preferred over the more conventional NOESY‐^1^H,^13^C‐HSQC one because it relies on the ^13^C dimension to separate NOE cross peaks rather than the ^1^H dimension. This approach is advantageous because the ^13^C^β^ resonances of the four cysteine residues of RALF8 are spread across a wider chemical shift range than the corresponding ^1^H^β^ resonances. Furthermore, in order to increase the resolution in the ^13^C dimension, we modified the evolution scheme for both indirect ^13^C dimensions to decouple ^13^C^β^ nuclei from their attached ^13^C^α^ and to reduce the ^13^C spectral window (12.5 ppm) so that only a narrow region centered around the cysteine ^13^C^β^ chemical shifts (^13^C offset set to 40.645 ppm) was detected. We used this modified pulse program to record a high‐resolution 3D ^1^H,^13^C,^13^C spectrum. After processing it with NMRPipe, careful examination of this 3D NOESY spectrum in NMRFAM‐SPARKY allowed us to identify clear NOE contacts between the ^1^H^β^ nuclei of C21 and C29 and between the ^1^H^β^ nuclei of C41 and C47, indicating that C21–C29 and C41–C47 are the correct pairing for the two disulfide bonds in RALF8.

**Figure 7:**
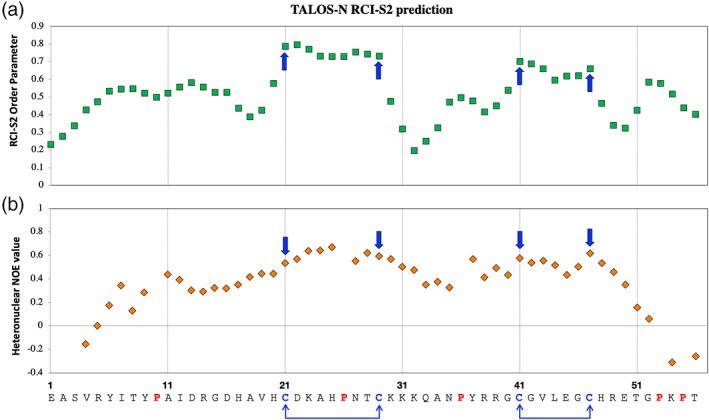
(a) RCI‐S^2^ index of RALF8 predicted by TALOS‐N using the chemical shifts of assigned backbone resonances and (b) ^1^H,^15^N‐heteronuclear NOE values for RALF8 backbone amides measured experimentally. The blue arrows point to the four cysteine residues that are present in the sequence of RALF8 (C21, C29, C41, and C47).

To get an understanding of the structural content of RALF8 in solution, we used the assigned chemical shifts of the backbone atoms as input to four secondary structure prediction programs: PECAN, TALOS‐N, GetSBY, and ncSPC. The results from these programs all agreed that RALF8 shows very little propensity for secondary structure (data not shown). Moreover, backbone order parameter S^2^ derived from random coil index (RCI‐S^2^) values predicted by TALOS‐N [Fig. 7(a)] indicates that the backbone of RALF8 is quite flexible in solution. Only two short stretches of amino acids exhibit a higher degree of order [Fig. 7(a)]. Interestingly, these are the two stretches of the sequence flanked by the four cysteine residues (C21–C29 and C41–C47). This observation is in agreement with our analysis of the ^1^H‐^15^N heteronuclear NOE spectra that show the same two stretches of amino acids having the backbone amides with the largest heteronuclear NOE values [Fig. 7(b)]. These results are also in agreement with the detected disulfide pairing: C21–C29 and C41–C47 [bottom of Fig. 7(b)].

**Figure 8:**
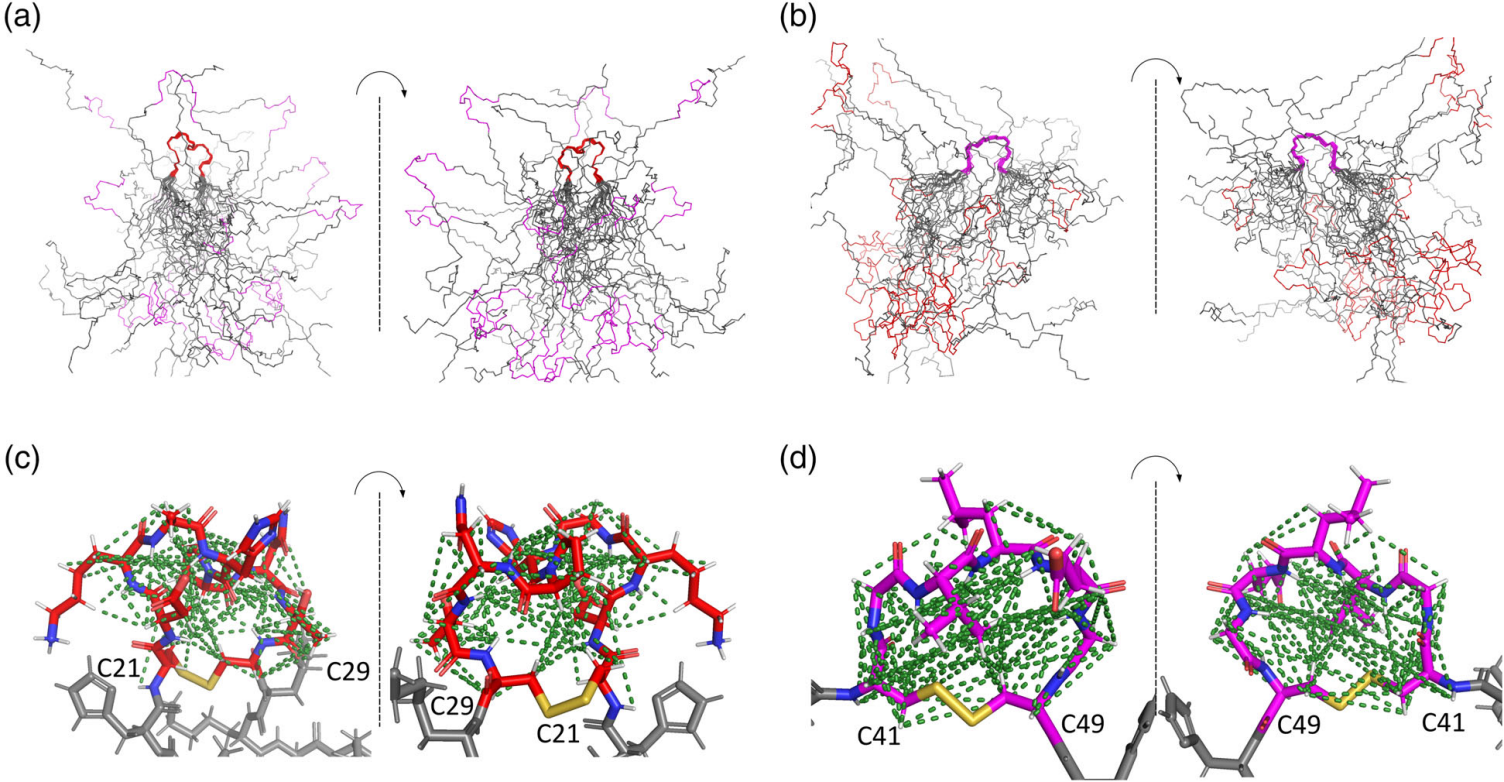
Solution structure of RALF8. The protein is dynamically disordered with the exception of two ordered loops that are held together by disulfide bridges. The C21–C29 loop (red) is more ordered than the C41–C47 loop (magenta), and the two ordered regions are independently ordered. (a) The 20 best structures for backbone atoms of RALF8 overlaid with reference to the structured residues in the C21–C29 loop. (b) The 20 best structures for backbone atoms of RALF8 overlaid with reference to the structured residues in the C41–C47 loop. (c) Structured C21–C29 loop region (white, hydrogen; blue, nitrogen; red double stick, oxygen; red, carbon; and gold, disulfide bond). (d) Structured C41–C47 loop region (white, hydrogen; blue, nitrogen; red double stick, oxygen; magenta, carbon; and gold, disulfide bond). The dotted lines represent NOEs used in determining the structures.

We used the NMR data as input to the Xplor‐NIH option of the PONDEROSA‐C/S program^25^ to determine models of the 3D structure of RALF8 (Fig. 8). The root mean square deviation (RMSD) between the backbone heavy atoms of all residues in the superimposed structures and those of the representative lowest energy structure was 12.8 Å, consistent with overall dynamic disorder. However, two loops between the disulfides (C21–29 and C41–47) were well defined with RMSD values of 0.6 and 0.7 Å for the backbone heavy atoms, respectively. Two sets of structural models, one superimposed on the structured region around C21–29 [see Fig. 8(a)] and the other superimposed on the structural region around C41–47 [see Fig. 8(b)], were deposited in the PDB under accession 6NU4.

## Conclusions

NMR spectroscopy easily discriminates between peptide preparations, such as RALF8, that are native and monodisperse and the ones that are misfolded, such as RALF1. Although both RALF1 and RALF8 are similar in polypeptide length and each contains four cysteine residues, the solubility and yield of the two peptides from *E. coli* differed to a large extent. Despite trying numerous vectors, bacterial fusions, and peptide production strategies, the NMR spectra of the RALF1 preparations were of uniformly poor quality. By contrast, we found it to be relatively straightforward to produce RALF8 in SUMO fusion‐based vectors and to isolate a soluble active product with intact disulfide bridges and a free glutamate at the N terminus. The fact that the recovery yield of RALF1 from *E. coli* was much lower than that of RALF8 suggests that RALF1 may be more susceptible to proteases than RALF8. Biological assays with *A. thaliana* seedlings revealed that RALF8, as previously shown for RALF1,^13^, ^15^ was potent to induce signal downstream events leading to root growth suppression. NMR confirmed the presence of two disulfide bonds in the peptide and showed that it its largely disordered in solution except for the regions near the disulfide bridges. The absence of a defined folded state of this peptide in solution suggests that additional structures may be induced only when interacting with a partner protein. Candidate proteins for such interactors include FERONIA, the receptor‐like kinase that mediates RALF1 action, as well as the possible coreceptors or chaperones, such as LLG1.^14^

## Materials and Methods

### *DNA cloning, production, and purification of RALF8 peptide*

*Arabidopsis thaliana* genomic DNA was used as a source to clone sequences corresponding to residues 72–120 of RALF1 (IniProt: Q9SRY3) and residues 27–82 of RALF8 (UniProt: Q1ECR9) into the T7 promoter‐based expression vector, pE‐SUMO (Kan) (LifeSensors). We also cloned the RALF1 gene into numerous vectors containing a variety of different N‐terminal fusion partners and tried protein purification strategies besides pE‐SUMO (Kan). However, none of the systems tested produced soluble active RALF1 peptides. Polymerase chain reaction (PCR) amplification and polymerase incomplete primer extension‐based techniques^26^ and KOD DNA polymerase (EMD Millipore) were used to amplify the RALF8 gene and insert it into the pE‐SUMO (Kan) vector. Chemically competent 10G *E. coli* cells (Lucigen) were transformed with ligation reaction and, after overnight growth of cells, recombinants were screened by PCR with 48‐base primers that anneal to 24 base regions in the 5′ and 3′ termini of the RALF8 gene. Positive recombinants were grown overnight at 37°C in 4 mL of 2YT (plus 50 μg/mL kanamycin), harvested, and used to purify expression plasmids for DNA sequencing analysis (DNASTAR Lasergene), and then archived and stored at −20°C.

The *E. coli*‐based production of RALF8 and RALF1 peptides was first optimized and screened at a small scale using 96‐well growth blocks, 0.5 mL of Luria‐Bertani (LB) growth medium per well, and *E. coli* B T7 SHuffle® expression strain (New England BioLabs). The T7 SHuffle strains have genetic mutations (*gor‐*, *trxB‐*, ahpC*, and cytoplasmic DsbC) that permit disulfide bond formation in proteins as they fold during translation in the *E. coli* cytoplasm.^27^ Chemically competent T7 SHuffle B cells were transformed with RALF8 pE‐SUMO (Kan) plasmid and grown on the LB plate (plus 50 μg/mL kanamycin and 1% glucose) overnight at 37°C. The next day, wells in a 96‐well growth block were filled with 0.5 mL of LB (plus appropriate antibiotics) and were inoculated with fresh colonies, and then the growth block was shaken at 37°C (250 rpm) until the optical density (OD) at 600 nm (OD_600nm_) reached about 1 (usually takes between 1 and 2 h). Isopropyl β‐d‐1‐thiogalactopyranoside (IPTG) was added to a final level of 1 m*M* to each well, and the growth block was shaken at 37°C for 4 more hours. The block was spun using a JS 5.9 rotor at 2000*g* for 5 min to harvest the cells for protein production analysis. The pelleted cells were suspended in 100 μL BugBuster™ (EMD Millipore), lysed, and analyzed by SDS‐PAGE on 4–20% gels (BioRad). The pE‐SUMO (Kan) plasmid system produces the RALF8 peptide fused to the C terminus of a SUMO domain (108 aa, 10.2 kDa) containing a six His‐tag at the N terminus.^22^, ^28^ The SUMO‐RALF8 peptide fusion was overproduced as a soluble product in *E. coli* that can be isolated using immobilized metal affinity chromatography (IMAC).

In the case of RALF1, we were unsuccessful in producing the native sequence of the soluble active RALF1 peptide at either small scale or large scale with any of the expression plasmids tested. However, the RALF1 peptide containing an N‐terminal His‐tag could be prepared at a large scale by using the Flexi pVP67K (N‐terminal His‐tagged vector similar to pVP68K but without MBP fusion)^21^ and Rosetta2 *E. coli* host expression cells. The RALF1 peptide formed insoluble inclusion bodies under the growth conditions used and required refolding before protein purification. The His‐tagged RALF1 peptide was unstable and highly susceptible to proteolytic degradation during purification.

We were able to produce the native sequence of RALF8 on a large scale. We began by picking individual colonies of RALF8 pE‐SUMO(Kan)/T7 SHuffle B and growing them in 1 mL LB medium at 37°C for 1–4 h. Next, these 1 mL starters were used to inoculate 100 mL of MDAG noninducing medium,^29^ and the culture was grown overnight at 25–30°C for about 18 h. To prepare ^15^N‐ and ^13^C, ^15^N‐labeled RALF8 peptide, 50 mL of overnight MDAG inoculum was added to 1 L of M9‐based medium supplemented with 2–4 g ^13^C‐glucose and 1 g ^15^N‐ammonium chloride (along with appropriate antibiotics), and the culture was grown at 37°C until the OD_600nm_ reached ~1.0.^22^ Prior to chemical induction, the temperature of the shaker was lowered to 25°C; 0.4 m*M* IPTG was added as the inducer, and the culture was grown for about 24 h. The *E. coli* cells were harvested by centrifugation at 4200 rpm for 20 min at 4°C, and the cell paste was stored for a long term at −80°C.

### *Isolation and purification of RALF peptides*

Frozen cell pastes of RALF1 and RALF8 were processed similarly: they were thawed and resuspended in 55–60 mL of lysis buffer [50 m*M* Tris pH 8, 500 m*M* NaCl, 10% ethylene glycol, 5 m*M* imidazole, 2% v/v NP‐40 detergent, and 1 m*M* phenylmethylsulfonyl fluoride (PMSF)] supplemented with 50 μg/mL RNase, 25 μg/mL DNase, and 100 μg/mL chicken egg white lysozyme. To break open the cells, a Misonix 3000 sonicator was used at 4°C with a program of 1 s on or 4 s off, at 55–65% power for a total of 15 min. The lysed cells were clarified before protein purification by centrifugation at 28,000 rpm for 30 min at 4°C. In contrast to RALF1 fused to SUMO, which produced inclusion bodies and insoluble peptide, RALF8 fused to SUMO was soluble under the growth conditions used, and the solution was loaded on to a 10 mL Qiagen IMAC column that had been equilibrated with IMAC buffer A (50 m*M* Tris pH 8, 500 m*M* NaCl, 10% ethylene glycol, 5 m*M* imidazole, and 1 m*M* PMSF). The RALF8 peptide was loaded onto an IMAC column at a flow rate of 1 mL/min, and the column was washed first with 10 column volumes of IMAC buffer A and then with 10 column volumes of IMAC buffer B (IMAC buffer A with 30 m*M* imidazole). His×6‐SUMO‐RALF8 was eluted with 30 mL of IMAC elution buffer (IMAC buffer A with 500 m*M* imidazole). The column fractions were analyzed by SDS‐PAGE to assess the purity and success of RALF8 peptide purification. To remove the SUMO fusion domain, 1 mg of SUMO protease (produced in‐house) was added to the purified His×6‐SUMO‐RALF8, and the mixture was dialyzed overnight in SUMO cleavage buffer (50 m*M* Tris, pH 8, 150 m*M* NaCl, 10% ethylene glycol, 5 m*M* imidazole, 1 m*M* PMSF, and 0.1 m*M* β‐mercaptoethanol). The usage of a low concentration of reductant ensured that the disulfide bonds in RALF8 were not broken during SUMO protease cleavage of the SUMO domain from the RALF8 peptide. The SUMO domain was removed by subtractive IMAC, and the flow‐through contained the native sequence of RALF8. The RALF8 peptide was concentrated on spin concentrators (Sartorius Vivaspin and Millipore) and polished further by cation exchange chromatography on a 5 mL GE SP cation exchange column; the peptide was bound to the column and then equilibrated with buffer A (50 m*M* MES, pH 6) and finally eluted by a salt gradient with elution buffer B (50 m*M* MES, pH 6, 1*M* NaCl). For RALF1, cell growths were harvested and lysed by the method used for RALF8. However, the RALF1 peptide was insoluble, and the inclusion bodies were harvested and washed with lysis buffer (with and without NP‐40 detergent, centrifuged at 18,000 *g* for 15 min), and denatured overnight in 60 mL 6 *M* guanidinium chloride containing 1 m*M* dithiothreitol. The denatured RALF1 peptide was clarified by centrifugation (28,000 rpm for 30 min) and then refolded by rapid dilution (dropwise) into 1 L of 50 m*M* Tris, pH 8, 500 m*M* NaCl, 10% ethylene glycol, 2 m*M* reduced glutathione, 1 m*M* oxidized glutathione, and 1 m*M* PMSF. After storing overnight at 4°C, the solution was applied to an IMAC column to isolate His‐tagged RALF1.

### *Mass spectrometry of RALF8*

Purified RALF8 samples were analyzed using an Agilent liquid chromatography mass spectrometry–time‐of‐flight (LC/MSD‐TOF) set in positive mode. The mass measurement of the purified ^15^N‐labeled RALF8 peptide using an ESI‐TOF‐MS instrument detected peptide ions with charges ranging from 5+ to 12+ (Fig. 3), reflecting the protonation of multiple Lys and Arg residues present in this peptide. Deconvolution of these charged states led to an average mass of 6311.8 (Fig. 3). We used the MS isotopic algorithm at the Protein Prospector site (http://prospector.ucsf.edu/prospector/mshome.htm) to model the level of ^15^N incorporation. Assuming that all the peptide molecules in the analyte are uniform and contain two disulfide bridges, the elemental composition for the oxidized form of fully ^15^N labeled RALF8 is C265 H418 ^15^N87 O80 S4, which predicts a theoretical nominal mass of 6311.77016, which represents 19.56% of the total. Although this comparison of the observed average mass assigned by an instrument software and the theoretical nominal mass are reasonably close, to obtain a more accurate estimation of ^15^N incorporation to RALF8 peptide, we used the *m*/*z* value of the most abundant species, the 9+ charge state [M + 9H]^9+^. The mass resolution for the modeling was set to 10,000, which matches that of the ESI‐TOF‐MS instrument. This approach yielded 93% as the average level of ^15^N in the ^15^N‐labeled sample of RALF8.

### *Bioassay of RALF8*

For the cytoplasmic calcium mobilization assay [Fig. 4(a)], *A. thaliana* seedlings expressing the calcium reporter, aequorin,^30^ were grown on half‐strength Murashige–Skoog (½ MS) medium for 5 days. Single seedlings were incubated in 200 μL ½ MS media supplemented with 2.5 μ*M* coelenterazine cp (Sigma‐Aldrich) in the dark for 16 h. RALF8 was added to the medium immediately before measuring bioluminescent emission. Luminescent signals were measured with a plate reader (Tecan) for 150 s. The total luminescence emission reflecting an increase in calcium concentration was calculated by summing signals obtained during 150 s. For a negative control, a volume of water or buffer equivalent to the RALF8 solution was added.

For the RALF8 root growth inhibition assay [Fig. 4(b,c)], wild‐type (Columbia ecotype) and *fer‐4* knockout mutant seedlings were grown for 3 days on ½ MS solid medium and then transferred to 500 μL ½ MS liquid medium containing 0, 1, 2, or 5 μ*M* RALF8 peptide. Seedlings were further grown for 2 days. Images were captured using an office scanner (Epson), and root length was quantified using ImageJ software.

### *Preparation of NMR samples*

Freshly prepared samples of ^15^N‐ and ^13^C/^15^N‐labeled RALF8 and RALF1 were exchanged by dialysis into a solution containing 20 m*M* sodium phosphate buffer, pH 6.0, 200 m*M* sodium chloride, 0.05% sodium azide, and 10% D_2_O (Sigma–Aldrich); the final concentration of each protein was ~1.0 m*M*. Samples used for NMR spectroscopy (300 μL) were enclosed in 5‐mm susceptibility‐matched Shigemi NMR tubes (Shigemi, Allison Park, PA).

### *NMR data collection*

All NMR spectra were acquired using Varian VNMRS (Agilent Technologies, Santa Clara, CA) spectrometers operating at 600 or 800 MHz (^1^H); each was equipped with a cryogenic triple‐resonance probe. The temperature of the sample was regulated at 298 K for all the experiments. 2D ^1^H,^15^N‐HSQC and 3D HNCACB, 3D CBCA(CO)NH, 3D HN(CA)CO, and 3D HNCO spectra were collected to enable backbone assignments. 2D constant‐time ^1^H,^13^C‐HSQC and 3D C(CO)NH, 3D HBHA(CO)NH, and 3D H(C)CH‐TOCSY spectra were acquired to enable assignment of the remaining ^1^H and ^13^C resonances in the aliphatic spectral region. Finally, for structure calculations, 3D NOESY ^15^N‐HSQC and 3D NOESY ^13^C‐HSQC data sets were collected, both using a 200 ms mixing time. The acquisition parameters of all 3D recorded spectra are reported in Table 2. All 3D spectra, except for the 3D NOESY ^13^C‐HSQC, were recorded using nonuniform sampling (NUS) at a sampling rate of ~40%. All spectra were processed using NMRPipe,^31^ and the 3D spectra recorded using NUS were reconstructed and processed using the SMILE package^32^ included in NMRPipe.

**Table 2:** Experimental Details for 3D NMR Spectra Collected for Backbone and Side Chain Assignments and Structure Calculations^a^

Experiment	Number of scans	Spectral window (ppm) ^1^H × ^13^C(^1^H^#^) × ^15^N(^13^C^&^)	Complex points ^1^H × ^13^C(^1^H^#^) × ^15^N(^13^C^&^)	Offset (ppm) ^1^H × ^13^C(^1^H^#^) × ^15^N(^13^C^&^)
HNCACB	16	16 × 59.7 × 23	1024 × 96 × 48	4.76, 44.8, 117.5
CBCA(CO)NH	16	16 × 59.7 × 23	1024 × 72 × 36	4.76, 44.8, 117.5
HN(CA)CO	24	16 × 10 × 23	1024 × 48 × 48	4.76, 175.4, 117.5
HNCO	8	16 × 10 × 23	1024 × 48 × 48	4.76, 175.4, 117.5
C(CO)NH	16	16 × 66.7 × 23	1024 × 72 × 48	4.76, 41.9, 117.5
HBHA(CO)NH	16	16 × 6.25^#^ × 23	1024 × 72^#^ × 52	4.76, 4.76^#^, 117.5
H(C)CH‐TOCSY	8	16 × 5.75^#^ × 66.7^&^	1024 × 64^#^ × 64^&^	4.76, 4.76^#^, 41.9^&^
NOESY ^15^N‐HSQC*	16	16 × 11^#^ × 23	1024 × 120^#^ × 46	4.76, 4.76^#^, 117.5
NOESY ^13^C‐HSQC	8	16 × 10^#^ × 66.7^&^	1024 × 72^#^ × 64^&^	4.76, 4.76^#^, 42^&^
^13^C‐HSQC NOESY ^13^C‐HSQC*	32	16 × 12.5 × 12.5^&^	1024 × 48 × 48^&^	4.76, 40.645, 40.645^&^

a Spectra were collected with cryogenic probes at 600 MHz (indicated by *) or 800 MHz (^1^H) at a regulated temperature of 298 K.

To establish the S–S bond configuration of RALF8, we recorded a 3D ^1^H,^13^C,^13^C‐spectrum using a modified ^1^H,^13^C‐HSQC‐NOESY‐^1^H,^13^C‐HSQC experiment (see description in Results and Discussion). This additional 3D NOESY spectrum was acquired using a 200 ms mixing time, with 1024 × 48 × 48 complex points in the ^1^H, ^13^C, and ^13^C dimensions, respectively, and 32 repetitions for each free induction decay (FID). The indirect ^13^C dimensions were centered to the ^13^C^β^ region of cysteine (40.645 ppm) and narrowed down to 12.5 ppm to maximize the resolution. This 3D spectrum was then processed using NMRPipe and analyzed with NMRFAM‐SPARKY.^33^

### *NMR peak assignments*

The processed 2D ^1^H,^15^N‐HSQC, 3D HNCACB, 3D CBCA(CO)NH, 3D HN(CA)CO, and 3D HNCO spectra were analyzed in NMRFAM‐SPARKY. For backbone assignments, the program APES^34^ was used for automated peak picking. After manual inspection, the peak lists from 3D and 2D spectra were submitted to the I‐PINE web server (http://i-pine.nmrfam.wisc.edu) for automated backbone assignments using PINE‐SPARKY.2 automation plugin (two‐letter‐code ep) in NMRFAM‐SPARKY. These automated assignments were then verified and extended by hand using the tools available in NMRFM‐SPARKY. The final backbone chemical shifts were then analyzed to estimate the structural content of RALF8 peptide in solution by running PECAN,^35^ TALOS‐N,^36^ GetSBY,^37^ and ncSPC.^38^ After the backbone assignments were completed, we collected additional spectra (2D constant‐time ^1^H,^13^C‐HSQC, 3D C(CO)NH, 3D HBHA(CO)NH, and 3D H(C)CH‐TOCSY) and used the “transfer and simulate assignments” tool available in NMRFAM‐SPARKY to extend the backbone assignments to the aliphatic portion of the side chains. We were thus able to identify 100% of these resonances. All assigned chemical shifts have been deposited in the BMRB^39^ (code 30565).

### *Dynamics*

To assess the dynamic properties of RALF8, we also ran experiments to measure the ^1^H‐^15^N heteronuclear NOE parameters for all assigned backbone amides. Two spectra, with (NOE spectrum) and without (reference spectrum) a 3 s ^1^H saturation pulse train, were recorded in an interleaved fashion with 1024 × 128 points in the ^1^H and ^15^N dimensions, respectively, with 32 scans per FID. The spectra were then processed using NMRPipe and analyzed with the Peak Intensity Analysis tool in NMRFAM‐SPARKY. The ^1^H‐^15^N heteronuclear NOE values were extracted by calculating the height ratios of the corresponding peaks from the spectra recorded with and without ^1^H saturation.

### *Structure calculations*

An NMR‐STAR 3.1 chemical shift file was generated by a plugin of NMRFAM‐SPARKY and used as input to the PONDEROSA client program along with the raw 3D ^15^N‐edited NOESY and ^13^C‐edited aliphatic NOESY spectra. Tolerances were set to 0.025, 0.35, and 0.35 ppm for ^1^H, ^13^C, and ^15^N, respectively. The PONDEROSA program includes an automatic NOE cross peak detection module connected to a visual noise level selector in NMRFAM‐SPARKY.^37^ The 3D structure calculation invoked the AUDANA^40^ option, which automatically generated distance restraints, and TALOS‐N, to obtain dihedral angle restraints. The PONDEROSA client program then submitted the structure calculation job to the PONDEROSA web server. Subsequently, we used the “Distance Constraint Validator” and “Angle Constraint Validator” of the Ponderosa Analyzer to validate the constraints. Constraints were visualized with PyMOL, and the corresponding NOE cross peaks were visualized synchronously with NMRFAM‐SPARKY. After constraints were carefully verified, they were submitted to the PONDEROSA web server using the *Constraints‐Only X* option, which utilized Xplor‐NIH to carry out high‐temperature dynamics, simulated annealing, and low‐temperature dynamics. A total of 914 distance constraints (832, 66, and 16 constraints for short‐, medium‐, and long‐range, respectively) and 20 dihedral angle constraints (10 *φ* angles and 10 *ψ* angles) were used for structure calculation (Table 3). From a total of 100 calculated structures, we selected the 20 lowest energy structures calculated using the *Final Step with Explicit Water Refinement* option.

**Table 3:** Statistics From the RALF8 Structure Calculations^a^

Considered regions	1–56	21–29	41–47
Distance constraints			
Short range |(*i* − *j*)| ≤ 1	832	123	124
Medium range 1 < |(*i* − *j*) | < 5	66	31	23
Long range |(*i* − *j*)| ≥ 5	16	12	4
Total	914	166	151
Dihedral angle constraints			
*φ*	10	7	2
*ψ*	10	7	2
Total	20	14	4
Average RMSD to the representative coordinates			
Backbone heavy atoms N, C^α^, C^β^, and C^o^	12.81 ± 2.47	0.55 ± 0.28	0.70 ± 0.37
All heavy atoms	12.93 ± 3.10	1.23 ± 0.25	0.77 ± 0.33
PROCHECK *Z* scores (*φ* and *ψ*/all)	−3.11/–3.37	−1.06/–1.95	−4.52/–6.80
Ramachandran plot summary from PROCHECK (%)			
Most favored	72.8	83.1	72
Additionally allowed	18.5	16.9	19
Generously allowed	5.7	0	8
Disallowed	3	0	1
MolProbity Clashscore/*Z*‐score	3.05/1.00		
Ramachandran plot summary from MolProbity (%)			
Most favored	83.4	100	67.1
Allowed	9.4	0	20
Disallowed	7.1	0	12.9
PONDEROSA pseudopotential *E* (kJ/mol)	2169.08 ± 44.93		
Violations			
Distance constraints [>0.5 Å]	0		
Dihedral angle constraints [>5°]	0		
van der Waals constraints [>0.2 Å]	0		

a Structural qualities for three different regions were evaluated using PSVS 1.5 (http://psvs‐1_5‐dev.nesg.org).
